# Relationships between proactive personality and creativity: Mindsets and golden mean thinking as parallel mediators among Chinese third language students

**DOI:** 10.3389/fpsyg.2022.969209

**Published:** 2022-11-03

**Authors:** Weipeng Deng, Yanjing Dai, Yuhong Gao, Rongxin Lin, Fei Lei, Lin Lei

**Affiliations:** ^1^School of Foreign Studies, South China Normal University, Guangzhou, China; ^2^School of Management, Guangdong University of Technology, Guangzhou, China

**Keywords:** proactive personality, creativity, growth mindset, fixed mindset, golden mean thinking, third language (L3) learners, Chinese students, positive psychology

## Abstract

Third language (L3) learners have great potential in developing creativity; however, the factors affecting L3 learners’ creativity have received little attention. This study investigated the relationships between proactive personality, three different thinking patterns (i.e., growth mindset, fixed mindset, and golden mean thinking), and creativity among L3 learners. The participants were 220 Chinese students who attended an obligatory L3 course in college. The results showed that proactive personality, growth mindset, golden mean thinking, and creativity had significant intercorrelations. Moreover, the role of growth mindset and golden mean thinking as mediators of the proactive personality and creativity relation was supported, and the mediating effect of growth mindset was larger than that of golden mean thinking. However, the fixed mindset did not show a significant indirect effect on the path from proactive personality to creativity. This is the first research to treat growth mindset, fixed mindset, and golden mean thinking as mediating variables on the path from proactive personality to creativity, particularly demonstrating that golden mean thinking, which is specific to Asian students and located midway between a growth mindset and a fixed mindset, can cultivate creativity. Some suggestions for fostering creativity in L3 students were also included in this study.

## Introduction

China is increasingly interacting with foreign countries under the trend of globalization. The success of the World Expo, the Belt and Road Initiative, and the 2022 Winter Olympics has stimulated Chinese students’ interest in learning foreign languages (FLs), especially learning a second FL (i.e., third language, L3). In the past decade, the number of L3 learners in China is increasing and China plays a key role in promoting and sustaining L3 teaching and learning (Gao and Zheng, 2019). For instance, China had the largest number of Japanese language learners (1.046 million) in 2012 (Lv et al., 2017) and there were, according to Ministry of Education (MOE), 70.35 million Chinese students studying abroad in 2019 (Ministry of Education of the People’s Republic of China., 2020). It is also noteworthy that many Chinese students list non-English speaking countries (e.g., France, Germany, Italy, etc.) as the top destinations (Chen and Padilla, 2022). However, in contrast to considerable research studies on L3 learners in western context, there have been only a few studies that address L3 learners in Chinese context (Cenoz, 2003). Therefore, more attention should be paid to L3 learners in China.

Creativity is a culturally bound phenomenon rather than a simple metal process (Csikszentmihalyi, 1998), influenced by the different cultures of East and West. One of the most concise, coherent, integrated, and empirically testable aspects of variation between Eastern and Western cultures lies in individualism-collectivism (Kim et al., 1994). As a great and long-standing part of collectivism, golden mean thinking makes a tremendous impact on Chinese education (Yuan et al., 2021). In studies with Chinese people, two viewpoints on the effect of collectivism (including golden mean thinking) on creativity have been formed: one argues that collectivism has a negative effect on creative ideas generation (Wang et al., 2009), while the other suggests that golden mean thinking facilitates innovative behavior (Yao et al., 2010). Thus, it is necessary to verify the impact of golden mean thinking on creativity in more diverse populations.

For a long time, Asian students’ creativity have been deliberately ignored by its social value system. Students in collectivist countries (e.g., Iran and China) are asked to place more emphasis on being in line with others, whereas American teachers strive to foster critical thinking and creativity and focus more on students’ unique qualities (Hudson and Hoffman, 1993). Kharkhurin and Samadpour Motalleebi (2008) also found in a survey that students in individualist countries show greater ability in divergent thinking than Asian students. Therefore, more attention needs to be paid to the creativity of students (e.g., L3 learners) in collectivist contexts (e.g., China) in order to explore the factors that influence creativity in this group and thus develop effective enhancement strategies.

### Foreign languages learners’ creativity

Creativity comprises the abilities that are most characteristic of creative people (Guilford, 1950) and leads to the production of novel and useful ideas by an individual or a small group of individuals working together (Amabile, 1988). Creativity is important both at the individual and societal levels. According to Amabile (1988), creative people tend to break perceptual sets, explore new cognitive pathways, leave response possibilities open as long as feasible, suspend judgment, use wide categories in storing information, remember accurately and break out of performance scripts. At the societal level, creativity is not only important for new scientific findings, new movements in art, new inventions, and new social programs but also indispensable for the economy by creating new products and jobs (Sternberg and Lubart, 1999).

For FL learners, creativity is equally important because bilingual/multilingual skills are closely related to almost all aspects of creativity (Fürst and Grin, 2018, 2021). Furthermore, researchers have found that FL learners with higher creativity tend to show a greater degree of openness to experience, cognitive flexibility, self-esteem, resilience, and positive emotions (Chen and Padilla, 2022), all of which are psychological factors that contribute to FL learning. Therefore, creativity is generally considered to be beneficial, useful, and valuable for FL learners.

Given the impact of creativity as a personal trait (Sharp, 2008) on personal development and FL learning, it is important to investigate the factors that may contribute to it. In the field of foreign language acquisition (FLA), some researchers have explored factors that affect second language (L2) learners’ creativity, such as verbal humor and optimism (Forman, 2011; Lin Lei and Lei, 2022). However, there is a lack of research on the creativity of FL learners, especially the influence of positive psychological factors (e.g., proactive personality) on the creativity of this group.

In addition, L3 acquisition, which is the acquisition of a non-native language by learners who have previously acquired or are acquiring two other languages, is an important part of FLA but has received less attention than L2 acquisition (Cenoz, 2003). Therefore, it is important to focus more on the creativity of L3 learners. However, few studies have explored the factors that may influence the creativity of L3 learners.

Previous studies have suggested that individuals’ proactive personality, growth mindset, and fixed mindset are correlated with their creativity (Kim et al., 2009; Intasao and Hao, 2018). However, even though they are individuals’ important traits and behaviors, these variables are rarely studied in relation to L3 learners’ creativity. Additionally, considering the characteristics of L3 learners in Asia, this study introduces golden mean thinking, a classical Confucian thought, to explore the possible role of this indigenous mindset on the creativity of Asian FL learners. In conclusion, this study goes beyond previous studies and puts proactive personality, growth mindset, fixed mindset, golden mean thinking, and creativity in one system to explore and compare the impact of the dimensions of Asian university students’ (e.g., Chinese university students) thinking on creativity in a more comprehensive way.

### Proactive personality and creativity

Proactive personality is a positive trait that encourages people to respond to problems in a proactive manner (Bateman and Crant, 1993). Bateman and Crant (1993) conceived the proactive personality as one that is generally unfettered by situational factors and influences environmental change, such as by adjusting to an unsatisfactory working situation (Hirschman, 1970). People with a proactive personality look for opportunities, take initiative and continue until they achieve their goals. In contrast, people without a proactive personality failed to scan for, let alone seize, opportunities to change the *status quo* (Crant, 1995).

Several studies have examined the correlation between proactive personality and creativity. Highly proactive individuals adapt to their surroundings and grab the opportunity to address difficulties or enhance their performance with innovative ideas and new approaches (Bateman and Crant, 1993; Seibert et al., 2001; Kim et al., 2009). Moreover, employees with a proactive personality tend to build energy, inspiration, and motivation, making them more engaged and creative at their work and able to recognize issues and devise solutions to them (Crant, 2000; Bakker et al., 2018; Zhang et al., 2021; Karimi et al., 2022). However, it should be noted that most studies on the relationship between proactive personality and creativity have focused on only the work and organizational field. In addition, although proactive personality has been discussed in the field of education (Zhu et al., 2017; Yang et al., 2018; Chen et al., 2021), little research in this field has investigated the relationship between proactive personality and creativity (Gao et al., 2019), and even less work has been completed in the field of language acquisition (e.g., L2 and L3 acquisition).

### Mindsets as mediators

#### Proactive personality and mindsets

Mindsets are individuals’ beliefs about whether their intelligence is malleable, meaning that some conceive their intelligence as a malleable quality, whereas others think of theirs as a fixed entity (Dweck and Leggett, 1988). Dweck (1999) further explained the former individuals as having a growth mindset or an “incremental theory” of intelligence. This group of people holds the belief that their intelligence can grow through practice and efforts. The other group, however, possesses a fixed mindset or an “entity theory” of intelligence, believing that their intelligence is set within themselves and hardly experiences changes.

The prominence of mindsets in the domains of educational and social psychology is related to its influence on and reflection of individual behaviors. It has been shown that whether individuals think of their abilities as fixed or malleable can lead to different outcomes (Dweck and Leggett, 1988; Hong et al., 1999; Intasao and Hao, 2018). Individuals with a fixed mindset tend to attribute their successes and failures to the existence or absence of certain abilities; those with a growth mindset, however, were likely to associate the results with their efforts (Hong et al., 1999; Haimovitz and Dweck, 2017; Intasao and Hao, 2018).

The direct relationship between proactive personality and mindsets has also been explored. Raub (2010) found that individuals with high proactivity are more motivated to view their intelligence as malleable, which is known as the growth mindset, and hence are more inclined to engage in proactive learning behavior. Conversely, individuals who are low proactive tend to regard their intelligence as a fixed entity, which is known as the fixed mindset, and focus on only their current skillset (Dweck and Leggett, 1988). In addition, research has found a growth mindset is positively associated with employees’ work engagement, and specifically, a proactive personality is highly related to work engagement for individuals with a growth mindset (Caniëls et al., 2018). However, research on the direct and indirect relationship between proactive personality and mindsets is still limited, especially in L3 learning.

#### Mindsets and creativity

It is noteworthy that only until the recent decade has the relationship between individuals’ mindsets and creativity received increasing attention. For instance, a growth mindset guides individuals to solve problems more creatively and originally, while those guided by a fixed mindset are less creative (O’Connor et al., 2013; Intasao and Hao, 2018). According to Intasao and Hao (2018), a fixed mindset is negatively related to creative performance, and a high creative growth mindset tends to be connected with desirable creative performance. Such finding also conforms to what Li et al. (2021) have discovered: students adopt more creative behaviors through learning, working on, and achieving new matters.

The effect of mindsets on creativity can also be explained by individuals’ feedback-seeking behaviors. Previous research has found that a growth mindset leads individuals to welcome feedback from various sources, while a fixed mindset drives individuals away from feedback because it may carry setbacks (Dweck, 1999; Devloo et al., 2011; McClendon et al., 2017; Waller and Papi, 2017; Papi et al., 2018; Cutumisu and Lou, 2020). As individuals can develop creative thoughts through various feedback sources (Stobbeleir et al., 2011; Lee and Kim, 2021), a growth mindset, which leads individuals to seek feedback, is conducive to fostering their creativity.

Although previous research has touched on the two mindsets’ impact on individuals’ creative performance, there is still space for deeper studies of the relationships among creativity, growth mindset, and fixed mindset. Therefore, we hope to investigate the existing research findings through empirical studies and provide insights for future studies on such relationships.

### Golden mean thinking as a mediator

#### Proactive personality and golden mean thinking

The term “golden mean,” i.e., the “ZhongYong (中庸),” originated from Confucianism. “Zhong” means moderation and appropriateness; “Yong” means perseverance and principles (Wu et al., 2020). It was initially regarded as the highest moral standard. Over time, it gradually evolved into a Chinese way of dialectic thinking as a metacognitive process (Chiu, 2000), having the connotation of “master the extremes, but deploy the mean” (执两端而允中) (Gao et al., 2022, p. 5). Wu and Lin (2005) defined golden mean thinking as “thinking about the same thing from multiple perspectives and choosing a behavior that can take care of the self and the overall situation” (p. 258). Wu and Lin (2005) also divided golden mean thinking into “multi-thinking,” “harmonious thinking” and “integration thinking” (p. 258). Integration thinking involves the integration of external information and inner thoughts and has the most significant correlation with creativity among the three types of thinking (Zhang and Gu, 2015). People with golden mean thinking emphasize peace as the most important thing, for which they reduce friction and conflict with others (Zhang and Gu, 2015). Moreover, they carefully observe the consequences that their actions may bring to others and the overall situation and formulate appropriate plans based on the actual situation (He, 2009). Golden mean thinking embodies an eclectic and holistic perspective, which suggests that golden mean thinking might be somewhere between a growth mindset and a fixed mindset.

However, it is noteworthy to point out that golden mean thinking is “not a simple compromise formula of binary contradictory view, but based on the independent perspective of a “third pole,” integrating and transcending of the two ends of a contradiction” (Ma, 2021, p. 28). Influenced by the golden mean thinking, Chinese students tend to speak and act cautiously, avoiding offending others and losing his or her own faces, and their most frequent classroom experience is listening to teachers (Liu and Littlewood, 1997). However, researchers have reached a consensus that despite different definitions and scales (Wu and Lin, 2005; Chang and Yang, 2014), golden mean thinking could facilitate one’s cognitive operation, enabling individuals to interact with the external world from a multidimensional and integrated perspective.

Many studies have shown that high golden mean thinking and high proactive personality can guide individuals to find and capture opportunities, thus improving interpersonal relationships and increasing creativity (Crant, 2000; Zhang et al., 2001; Kim et al., 2009; Zhang and Gu, 2015). These shared functions reflect an overlap between proactive personality and golden mean thinking, but there are few studies on the degree of the overlap or the direct relationship between these two factors. Qing and Liu (2014) confirmed that golden mean thinking plays an important role in mediating employee proactive personality and inspection behavior. Recently, it was supported that people with high proactive personality have greater ability in comprehending information from multiple perspectives and carrying out actions that is good for the organization (Ye et al., 2020), which is in line with the multidimensionality and harmony required by golden mean thinking. However, more studies need to be performed to explore the impacts of proactive personality on golden mean thinking in various groups.

To date, there is no study on the relationship between golden mean thinking and proactive personality in the field of FLA, but in a highly dynamic and hypercompetitive world, people with proactive personality are more capable of engaging in proactive behavior to determine creative solutions (Qing and Liu, 2014). In particular, Chinese students had long been influenced by golden mean thinking, so they were more likely to designate “appropriate” programs according to the actual situation (He, 2009). Thus, to better understand Chinese FL students’ relationship between proactive personality and golden mean thinking should not be ignored.

#### Golden mean thinking and creativity

Golden mean thinking is the Confucian doctrine of the mean, which encourages individuals to take a holistic perspective on situations before making decisions rather than acting upon impulse (Yao et al., 2010). Zhang and Gu (2015) pointed out that golden mean thinking adheres to management methods of focusing on the overall situation and collective interests, adjusting behavior according to the situation, avoiding unnecessary conflicts, and increasing the harmonious atmosphere of the organization, which helps develop positive moods. Moreover, many studies have corroborated that positive moods are related to higher creativity (Vosburg, 1998; Davis, 2009). Therefore, it can be inferred that golden mean thinking may be able to influence one’s creativity through moods.

In addition, research has shown that to achieve true and continuous innovation, individuals should follow the principle of moderate innovation, i.e., to insist on maintaining principles with flexibility, instead of following the old-fashioned way or going to extremes (Liu and Tan, 2003). Zhang and Gu (2015) confirmed that there is a significant positive correlation between modest thinking and employee creativity. Furthermore, golden mean thinking encourages learners to share their experiences with others (Liao and Dong, 2015), and information sharing is the key factor in stimulating creativity (Song et al., 2010). Recently, it was found that people with high golden mean thinking are ready to generate creative ideas and seek redress in a complicated situation due to their inclusiveness and willingness to collaborate with others (Wu et al., 2021). The above studies provide further evidence that the cultivation of golden mean thinking could be helpful in improving creativity.

Overall, golden mean thinking still has strong vitality in today’s innovative society, and it is worthy of continuous and in-depth study. Nevertheless, several studies have had a different understanding of golden mean thinking, holding that it hinders the cultivation of creativity because golden mean thinkers tend to be self-effacing, conservative, and complacent (Yao et al., 2010; Leung and Morris, 2011). Therefore, more empirical studies are needed to verify the positive/negative effect of golden mean thinking on creativity. Meanwhile, existing studies have mostly focused on the fields of moral education and business management, whereas few have focused on the relationship between college students’ (e.g., FL students) golden mean thinking and creativity, leaving much research space.

## Research aim and hypotheses

Previous studies have supported that L3 learning enhances learners’ creativity in various way. Successful multilingual learners have to keep a highly creative state while learning L3 because they need to frequently switch between languages, using their reasoning skills and flexible thinking to grasp the meaning (Cummins, 1976), which in turn enhances cognitive flexibility and creativity (Lei and Lei, 2022). In addition, researchers found that learning L3 could reduce communicative anxiety and FL anxiety (Dewaele et al., 2008), as well as improve their confidence (Thompson and Khawaja, 2015) and FL enjoyment (Ergün and Demirdağ, 2022), which are found beneficial to facilitate the generation and cultivation of creativity (Vidgren, 2016; Chen and Padilla, 2022). Furthermore, L3 learners are often immersed in a multilingual atmosphere, which facilitates viewing the world from various cultural perspectives and gaining cross-linguistic and cross-cultural experiences. The diversity of experiences helps shape divergent thinking and thus strengthen creativity (Mednick, 1962; Vidgren, 2016). It is thus evident that L3 learners have their own emotional, cognitive, and experiential characteristics that are potential factors affecting creativity, and therefore it is necessary to further explore the mechanisms of creativity production in this group.

**FIGURE 1:**
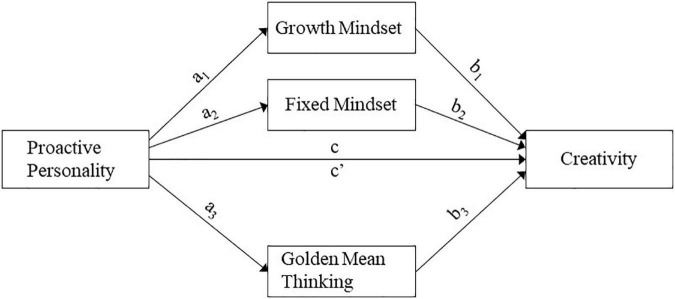
Conceptual mediation model of the mediating effects of growth mindset, fixed mindset, and golden mean thinking in the relationship between proactive personality and creativity.

Moreover, as discussed above, previous research suggested pairwise relationships between creativity with proactive personality, mindsets (growth mindset and fixed mindset), and golden mean thinking. However, we do not have a full picture of how these variables are related to each other in one system, especially for the L3 group. This research aims to explore how proactive personality, mindsets, and golden mean thinking predict L3 students’ creativity. In addition, the present research extends previous research by considering possible mediating roles that mindsets and golden mean thinking play in the relationship between proactive personality and creativity. The result will present an integrative picture of their relationships. From the findings of this study, a model can be formulated in which proactive personality is associated with mindsets, golden mean thinking, and creativity, while mindsets and golden mean thinking are associated with creativity. Figure 1 illustrates the conceptual mediation model regarding the mediating effects of growth mindset, fixed mindset, and golden mean thinking in the relationship between proactive personality and creativity. Thus, the following four hypotheses were formulated.

(1) Proactive personality is significantly correlated with mindsets (growth mindset and fixed mindset), golden mean thinking, and creativity;
(2) Mindsets (growth mindset and fixed mindset) and golden mean thinking are significantly correlated with creativity;
(3) Mindsets (growth mindset and fixed mindset) and golden mean thinking function as parallel mediating variables on the path from proactive personality to creativity;
(4) The effects of the mediators differ from each other.

## Materials and methods

### Participants and procedure

The participants were 220 native Chinese-speaking college students between the ages of 18 and 21 (*M* = 19.97, *SD* = 0.65). Most of the participants were females (93.64%), and the rest were males (6.36%). All the participants were English major students and were participating in an obligatory L3 course (i.e., French, German, Japanese, Korean, and Spanish) at a prestigious Chinese university. Among these participants’ L3, 48 learned French, 44 learned German, 70 learned Japanese, 37 learned Korean, and 21 learned Spanish. In this study, we focused on only FL learning in the context of L3 instruction.

The surveys were paper-based and self-administered. Before the surveys were distributed, invitations were sent to the teachers in charge of each L3 course (i.e., French, German, Japanese, Korean, and Spanish) to seek their consent for their students’ participation in this study. All six teachers gave their permission. Prior to distributing the surveys, we fully informed the students of the nature of this study, and they all agreed to participate. Then, the surveys were distributed during recess or the L3 classes with the support of the teachers in charge. During their participation, the students could withdraw from the study at any time at will.

Throughout the data collection procedure, all participants finished the surveys with paper and pen, and all the distributed surveys were retrieved immediately from the participants after they reported their completion. Then the retrieved paper-based surveys were allocated to half of the researchers for data entry using Office Excel; the other half, later, worked on comparing the typed-in data with the original and paper-based ones, so as to double-check the data accuracy for subsequent analysis.

After collecting the data, we examined all variables’ missing values through SPSS. Among all 220 surveys, missing data were rare (< 10%), and the serial mean was used for the replacement of the missing values (Tabachnick and Fidell, 2007).

### Measurement

#### Creativity

To assess the L3 learners’ creativity, we adopted the 13-item scale of Zhou and George (2001) (Cronbach’s α = 0.96). As this study revolved around the topic of L3 learning, items 3 and 4, related to the field of management, were deleted. The participants were required to indicate their levels of creativity on a 7-point Likert scale ranging from 1 (strongly disagree) to 7 (strongly agree). A sample item is “I suggest new ways to achieve goals or objectives.” Previous studies have corroborated the scale’s good reliability and validity (Shin and Zhou, 2003; Zhang and Bartol, 2010). Higher scores indicate higher levels of creativity. During the translation process, the method of translation and back-translation was adopted. One of the researchers first carried out the translated version of the 11 items, which was then back translated by another researcher of this study. Both researchers had a good command of Chinese and English. Then, all the researchers met to polish and adjust the Chinese version of the 11 items to prevent any discrepancies. The final step of the translation process involved inviting several FL professors to check the translated scale. Afterward, they were asked to comment on the translation to confirm its readability. Based on the data collected from the 220 participants, the instrument had good validity [χ^2^/*df* = 2.31; CFI = 0.94; TLI = 0.92; RMSEA (90% CI) = 0.08 (0.06–0.10); SRMR = 0.05] and adequate reliability (Cronbach’s α = 0.88).

#### Proactive personality

To measure the L3 learners’ proactive personality, we used the 10-item scale from Seibert et al. (1999) (Cronbach’s α = 0.86), which is a shortened Proactive Personality Scale (PPS) (Bateman and Crant, 1993). Then, the scale was translated into Chinese in accordance with the translation process above. The participants’ levels of proactive personality were tested on a 7-point Likert scale ranging from 1 (strongly disagree) to 7 (strongly agree). A sample item of the scale is “I am constantly on the lookout for new ways to improve my life.” The scale has exhibited good reliability and validity (Fuller and Marler, 2009). Higher scores suggest higher levels of proactive personality. After collecting the data, 4 items (items 6, 8, 9, 10) with factor loadings lower than 0.50 were dropped, which minimally affected the reliability of the scale. Finally, the collected data from the 220 participants indicated that the 6-item instrument had adequate validity [χ^2^/*df* = 2.07; CFI = 0.95; TLI = 0.92; RMSEA (90% CI) = 0.07 (0.02–0.12); SRMR = 0.04] and adequate reliability (Cronbach’s α = 0.72).

#### Mindsets

For the assessment of the L3 learners’ mindsets, we adopted two language mindset scales by Papi et al. (2019), which were originally adapted from Dweck’s Implicit Theories of Intelligence Scale (ITIS) for adults (Dweck, 1999). One scale was concerned with growth mindset, while the other was concerned with fixed mindset. Both scales have good reliability according to Papi et al.’s (2019) study (Cronbach’s α of Growth L2 Mindset = 0.93, *n* = 4; Cronbach’s α of Fixed L2 Mindset = 0.92, *n* = 4). Then, the scale was translated into Chinese according to the translation process above. On a 7-point Likert scale ranging from 1 (strongly disagree) to 7 (strongly agree), the participants were tested on the extent to which they possessed a growth L3 mindset or a fixed L3 mindset. For instance, items such as “You can always improve your language learning intelligence” were used to assess the participants’ level of growth L3 mindset, while a sample item for a fixed L3 mindset is “You have a certain amount of intelligence for learning other languages, and you can’t really do much to change it.” The original scale’s good reliability and validity were indicated (De Castella and Byrne, 2015). Higher scores suggest higher levels of either growth mindset or fixed mindset. Judging from the collected data, the instrument possesses good validity [χ^2^/*df* = 1.47; CFI = 0.98; TLI = 0.98; RMSEA (90% CI) = 0.05 (0.00–0.08); SRMR = 0.03], and its reliability is good (Cronbach’s α of Growth L2 Mindset = 0.80, *n* = 4; Cronbach’s α of Fixed L2 Mindset = 0.82, *n* = 4).

#### Golden mean thinking

To assess the L3 learners’ golden mean thinking, this study selected the subscale of integration thinking from the Zhong Yong Thinking Style Scale developed by Wu and Lin (2005). As Wu and Lin (2005) have corroborated that integration thinking in golden mean thinking is strongly correlated with individuals’ creativity, we extracted the subscale of integration thinking, which consists of 5 items and has been shown to have good validity by Wu and Lin (2005) (Cronbach’s α = 0.73). Because the subscale was originally written in Chinese, it was used directly without translation. The participants were asked to use a 7-point Likert scale ranging from 1 (strongly disagree) to 7 (strongly agree) to evaluate their levels of golden mean thinking. A sample item is “I try to come up with a universally acceptable opinion in occasions where opinions are in conflict.” Higher scores suggest higher levels of golden mean thinking. The data collected from the 220 participants proved that the instrument had adequate validity [χ^2^/*df* = 2.03; CFI = 0.98; TLI = 0.97; RMSEA (90% CI) = 0.07 (0.00–0.13); SRMR = 0.03] and adequate reliability (Cronbach’s α = 0.79).

## Data analysis

Data analyses were conducted by using SPSS 25.0 and PROCESS software in SPSS. The preliminary analysis included means, standard derivations, skewness, kurtosis, and correlations of the study variables (see Table 1). To examine normality, this study used Z-standardized values of skewness and kurtosis (i.e., Z_*skewness*_ and Z_*kurtosis*_) for proactive personality, growth mindset, fixed mindset, golden mean thinking, and creativity. According to Ghasemi and Zahediasl (2012), for samples larger than 200 with small standard errors, the Z-standardized values of skewness and kurtosis should be in the range of −2.58 to 2.58 to indicate normality. For Pearson’s correlation analysis, correlation coefficients of 0.10, 0.30, and 0.50 represent small, medium, and large effect sizes, respectively (Cohen, 1988).

**TABLE 1:** Means, standard derivations, skewness, kurtosis, correlations of the study variables.

Variables	M	SD	Z_skewness_	Z_kurtosis_	1	2	3	4	5
1. Proactive personality	5.08	0.73	2.41	–1.04	1				
2. Growth mindset	4.66	0.94	–0.71	2.22	0.56**	1			
3. Fixed mindset	4.17	1.07	–0.32	0.54	–0.01	−0.16*	1		
4. Golden mean thinking	5.62	0.73	–1.65	–0.30	0.50**	0.34**	–0.08	1	
5. Creativity	4.97	0.78	0.62	–0.52	0.73**	0.64**	–0.01	0.54**	1

*p < 0.05, **p < 0.01.

The mediation analysis was performed by PROCESS software, and Model 4 was used (Hayes, 2017). Since the participants were from the same grade and mainly female, age (SD = 0.65) and sex (less than 7% of the participants were males) would not be served as control variables. The direct and indirect effects were estimated with the bootstrap method, and the significance of the direct and indirect effects were determined by the 95% confidence interval (CI). The direct and indirect effects are significant when the 95% CI does not cover zero (Chen et al., 2019).

## Results

### Preliminary analysis

Table 1 shows the means, standard derivations, skewness, kurtosis, and correlations of the variables. The score of golden mean thinking (mean = 5.62) was close to 5.6 (seven-point scale), indicating that Chinese L3 students are strongly influenced by traditional culture and highlighting the importance of golden mean thinking as an influencing factor. Moreover, the scores of proactive personality, growth mindset, and creativity were greater than 4.6, indicating that Chinese L3 students have positive personality traits.

As presented in Table 1, the absolute values of Z_*skewness*_ ranged from 0.32 to 2.41, and the absolute values of Z_*kurtosis*_ ranged from 0.30 to 2.22, showing that the study variables were normally distributed (Ghasemi and Zahediasl, 2012; Hinton, 2014; Wen et al., 2018). In addition, the variance inflation factors (VIFs) of all independent and mediator variables were less than 3, indicating no multicollinearity problem; thus, subsequent correlation and mediation analyses could be conducted (O’brien, 2007; Wen et al., 2018).

The results of the correlation analysis in Table 1 indicate that, as predicted, proactive personality positively correlates with growth mindset, golden mean thinking, and creativity (all *p* < 0.01), and the sizes of correlations were large (*r* = 0.50–0.73). Moreover, the growth mindset and golden mean thinking showed moderate positive intercorrelations (*r* = 0.34, *p* < 0.01) and were strongly positively correlated with creativity (*r* = 0.54–0.64, *p* < 0.01). However, the fixed mindset was not significantly correlated with proactive personality, golden mean thinking, and creativity (all *p* > 0.05). The above results fully support Hypothesis 1, whereas Hypothesis 2 is partially supported.

### Mediation analysis

**FIGURE 2:**
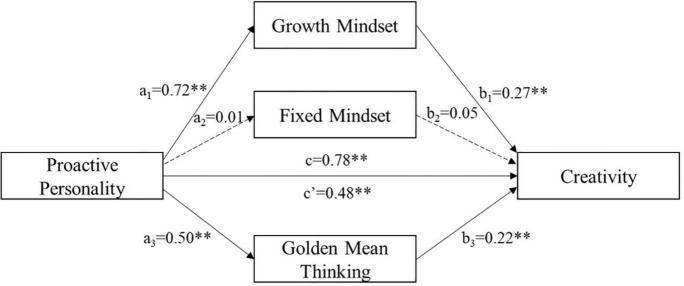
Mediation model linking proactive personality and creativity through growth mindset, fixed mindset and golden mean thinking.

As shown in Figure 2, proactive personality positively predicted the growth mindset (β = 0.72, *p* < 0.01) and golden mean thinking (β = 0.50, *p* < 0.01), presented by a_1_ and a_3_, respectively. Furthermore, the growth mindset (β = 0.27, *p* < 0.01) and golden mean thinking (β = 0.22, *p* < 0.01) both positively predicted creativity, presented by b_1_ and b_3_, respectively. However, proactive personality had no significant impact on the fixed mindset (*p* > 0.05), and the effect of the fixed mindset on creativity was also insignificant (*p* > 0.05). The above results provide preliminary evidence that the growth mindset and golden meaning thinking mediate the relationship between proactive personality and creativity.

The results in Table 2 indicate that the total effect of proactive personality on creativity was 0.78 (*SE* = 0.05, 95% CI = 0.69–0.88), the direct effect was 0.48 (*SE* = 0.06, 95% CI = 0.37–0.59), the total indirect effect was 0.31 (*SE* = 0.04, 95% CI = 0.22–0.41), and the ratio of the total indirect effect to the total effect was 38.91%. This result further demonstrates the existence of mediating effects and that the total mediating effect was strong (> 0.25) (Kenny, 2015), even though the direct effect was larger than the total mediating effect. This suggests that mediating variables play an important role in this model.

**TABLE 2:** Direct and indirect effects of proactive personality on creativity through growth mindset, fixed mindset, and golden mean thinking (three pathways for indirect effects).

	Estimated	*SE*	95% CI (Lower)	95% CI (Upper)	Relative effect
Total effect	0.78^a^	0.05	0.69	0.88	–
Direct effect	0.48^a^	0.06	0.37	0.59	–
Indirect effects	0.31^a^	0.05	0.22	0.41	38.91%
*Path 1:* Proactive personality ↓ Growth mindset ↓ Creativity	0.20^a^	0.04	0.13	0.28	24.99%
*Path 2:* Proactive personality ↓ Fixed mindset ↓ Creativity	0	0.01	-0.02	0.01	0
*Path 3:* Proactive personality ↓ Golden mean thinking ↓ Creativity	0.11^a^	0.03	0.05	0.17	13.98%

SE, standardized error; ^a^empirical 95% confidence interval (CI) does not include zero.

Specifically, as shown in Table 2, the indirect path from proactive personality through the growth mindset to creativity was significant, with an effect of 0.20 (*SE* = 0.04, 95% CI = 0.13–0.28). Similarly, the indirect path from proactive personality through golden mean thinking to creativity was significant, and the effect was 0.11 (*SE* = 0.03, 95% CI = 0.05–0.17). By comparing the two effect sizes above, it is clear that the indirect effect from proactive personality to creativity *via* the growth mindset (relative effect = 24.99%) is significantly larger than that *via* golden meaning thinking (relative effect = 13.98%). However, the indirect path from proactive personality through the fixed mindset to creativity was insignificant (95% CI = -0.02 to 0.01). Therefore, Hypothesis 3 is partly supported, and Hypothesis 4 is supported.

## Discussion

### Proactive personality and creativity in Chinese third language students

The purpose of this study was to examine the relationships among proactive personality, growth mindset, fixed mindset, golden mean thinking, and creativity among Chinese L3 learners. The above results reveal that positive personality, growth mindset, golden mean thinking, and creativity have positive intercorrelations. The growth mindset and golden mean thinking mediate the path from proactive personality to creativity, and the growth mindset outperforms golden mean thinking when these two significant mediators are compared. However, the fixed mindset fails to mediate the relationship between proactive personality and creativity.

According to the correlational analysis in Table 1, there was a positive correlation between proactive personality and creativity, which is consistent with the findings of previous research (Zhao and Gao, 2014; Kong and Li, 2018; Choi et al., 2021). When acquiring the third language, L3 learners are always curious about and interested in diverse cultures all over the world. Hence, L3 learners with higher proactive personality will take more initiatives to explore different cultures and learn L3 from the cultural experience. When faced with difficulties, they will draw on their own multicultural experience to find solutions from different perspective of cultural views. In this way, L3 learners’ multicultural experience, multilingualism, and proactivity can promote their creativity to tackle with the difficulties (Gong et al., 2012; Choi et al., 2021; Fürst and Grin, 2021).

### Growth mindset as a mediator

The current study was the first empirical investigation into the mediating role of the growth mindset on the paths from proactive personality to creativity. These results support the proposition that growth mindset could act as a mediating variable between proactive personality and creativity, which derived from the previous studies that growth mindset significantly correlated with proactive personality and creativity (Raub, 2010; Zhao et al., 2021). In the context of L3 learning in China, highly proactive L3 learners tend to take the initiative to seek opportunities to change their current learning situations and persist in looking for better solutions until their L3 proficiency has improved or their learning problems have been solved (Bateman and Crant, 1993).

Over time, after they have made progress in L3, they will have the multilingual ability to communicate with others. It’s convenient for them to toggle between languages and learn different meanings by using their reasoning ability and flexible thinking (Cummins, 1976). In this sense, L3 learners will believe in their ability to develop their learning skills and intelligence through efforts or perseverance (Dweck, 1999). Furthermore, the growth mindset that is integrated into their L3 learning will guide them to think with dynamic beliefs and facilitate their cognitive flexibility as well as creativity in L3 learning (Vidgren, 2016; Zhao et al., 2021; Chen and Padilla, 2022; Lei and Lei, 2022).

Moreover, the findings reveal that among Chinese L3 learners, growth mindset has a larger predictive effect on creativity than golden mean thinking and fixed mindset, which is a new contribution to L3 learning. Therefore, it is important for L3 learners to form a proactive personality and thus perceive a growth mindset to cultivate their creativity, and in the pedagogical process, growth mindset can serve as a particular focus to understand and foster L3 learners’ creativity.

### Fixed mindset as a mediator

The results also indicate that the fixed mindset fails to mediate the path from proactive personality to creativity, meaning that Chinese L3 learners’ fixed mindset rarely contributed to either the debasement or the fostering of their creative behaviors from proactive personality. A possible explanation is associated with the emotions that learners experience during their L3 learning. It was found that learning L3 could reduce learners’ communicative anxiety and FL anxiety (Dewaele et al., 2008), as well as improve their confidence (Thompson and Khawaja, 2015) and FL enjoyment (Ergün and Demirdağ, 2022), thus facilitating the generation and cultivation of creativity (Vidgren, 2016; Chen and Padilla, 2022). What’s more, positive affect also helps individuals to jump out of a fixed mindset (Haager et al., 2013). As such, the fact that L3 learners experience more positive emotions during L3 learning helps to impede a fixed mindset, showing that L3 learners’ fixed mindset may not pose great influence on their creative performance.

Such phenomenon may also be explained by previous studies, which have shown that the mechanism of the relationship between a fixed mindset and creativity is complex and can be influenced by the degree and type of performance goal. According to the implicit theory of Dweck and Leggett (1988), an individual who holds the belief that his or her intelligence is fixed (i.e., fixed mindset) tends to set up performance goals to “look smart” (i.e., to gain positive judgments or to avoid negative judgments), whereas the relationship between performance goals and creativity is unstable and may be positively or negatively correlated (Choi et al., 2014; Song et al., 2015; Du et al., 2020). Judging from this, the effect of fixed mindset on creativity through mediating variables (e.g., performance goals) may not be stable, either.

Moreover, some studies have revealed that creative role identity and self-efficacy mediate the relationship between performance goals and creativity (Song et al., 2015; Du et al., 2020), suggesting that fixed thinking may not directly influence creativity but indirectly influence it through other variables.

Thus, from the perspective of emotions, it is possible that there is no direct relationship between fixed mindset and creativity. Moreover, previous findings also indicate that when discovering a relationship between a fixed mindset and creativity, it is appropriate to consider other factors, such as performance goals, creative role identity, and self-efficacy. The above may explain why the fixed mindset was not associated with proactive personality or creativity in this study.

### Golden mean thinking as a mediator

The correlation table (Table 1) shows that golden mean thinking is positively correlated with the growth mindset but has no significant correlation with the fixed mindset, which implies that golden mean thinking tends to manifest itself more optimistically than negatively among Chinese L3 learners. People with proactive personalities tend to view problems in a positive light, which facilitates better achievement of the “neutral” mental state that is the embodiment of golden mean thinking (Bateman and Crant, 1993). It can be argued that proactive dispositions influence the degree of neutral thinking by affecting people’s attitudes toward problems. For L3 learners, a proactive personality could lead them to seek information and integrate it more objectively when learning different FLs, which may influence their golden mean thinking. The diversity of perspectives might subtly improve L3 learners’ degree of golden mean thinking, especially in a collectivistic context. In addition, a high proactive personality learner is more like a pathfinder (Leavitt and Bahrami, 1988) rather than a passive adaptor, i.e., a proactive learner is adept at identifying and solving problems, they are less susceptible to the environment and instead take initiative actions to change the environment until the goal is achieved (Bateman and Crant, 1993), which might in turn enhance cognitive abilities and thus improve the creativity.

Moreover, our findings show that L3 students with high golden mean thinking have higher creativity because they tend to deal with problems from multiple perspectives (Wu and Lin, 2005), considering both principles and innovation when learning an FL. This diverse thinking might occur more in a L3 learning context, since L3 learners are expected to flexibly shift their mindsets and strategies in learning different languages (Cummins, 1976). Additionally, golden mean thinking encourages students to seek various information and integrate this information, as well as to develop good interpersonal relationships (Crant, 2000; Kim et al., 2009), which helps them learn L3 in a productive, interactive, and pleasant way and to continuously summarize their experiences and improve their learning methods (Vosburg, 1998; Davis, 2009).

Furthermore, impressed by golden mean thinking, Chinese students tend to view the world from a holistic perspective and focus on society and human relations while making decisions (He, 2009). This integration thinking enables students to engage in more proactive and harmonious behaviors, which further improves the learning environment and boosts their creativity. The positive correlation between golden mean thinking and creativity may also be contributed to the inner consistency of golden mean thinking and L3 learning, i.e., they both impel people to view the world from various lens and combine different ideas. In view of the above findings, we tentatively consider that the ability to harmoniously cooperate with others and objectively integrate information, which seem more common among high proactive personality L3 learners, would improve one’s creativity through affecting his or her golden mean thinking in terms of multi-thinking ability, holistic thinking ability, positive behaviors and harmonious environment. Theoretically, the findings of meditation are validated by positive psychology theory. Positive personality traits (e.g., proactive personality) may affect and lead to positive emotions (Ruini, 2017), which, according to Fredrickson (2001), can broaden people’s mindsets (e.g., growth mindset and golden mean thinking) and generate persistent personal resources (e.g., creativity). In the FL setting, many studies considered positive psychology to play an important role in both FL learning and teaching (Dewaele et al., 2019; Macintyre et al., 2019; Chen and Padilla, 2022). Hence, it is necessary to foster students’ positive feelings to form a positive mindset and finally increase their creativity with proper positive psychology activities in the L3 context.

## Limitations

There are several limitations to this study. One limitation lies in the present study investigating only the aspect of integration thinking in golden mean thinking. Future studies should focus more on the other two, namely, multi-thinking and harmonious thinking, providing deeper insight into golden mean thinking. Moreover, a true growth mindset guides individuals to confront and learn from setbacks, and the process of tackling negative feelings caused by a fixed mindset will lead individuals to a true growth mindset during the learning journey (Dweck, 2015). Therefore, although the present study found the fixed mindset to have an insignificant effect in mediating the relationship between proactive personality and creativity, future studies should work to reveal ways to make use of a fixed mindset in the learning process. Additionally, considering that in our model, the growth mindset and golden mean thinking both played partial mediating effects, future studies should focus on other possible mediators related to creativity, such as feedback-seeking behavior (De Stobbeleir et al., 2011) and self-efficacy (Choi et al., 2021). What’s more, as our sample comprised only English major college students in a Chinese university, the applicability of our findings remains to be verified within a more diversified group of participants. Finally, it would be desirable to increase the sample size and use those more objective sampling methods to procure and maintain the soundness of research findings.

## Conclusion

This study first indicated that proactive personality, growth mindset, golden mean thinking, and creativity have positive intercorrelations, which supports the importance of cultivating Chinese L3 learners’ proactive personality, growth mindset, and golden mean thinking, aiming at promoting their creativity. Additionally, proactive personality positively predicts creativity. Furthermore, the mediation analysis shows that the growth mindset and golden mean thinking mediate on the path from proactive personality to creativity in parallel, and the indirect effect of the growth mindset is larger than that of golden mean thinking. However, the fixed mindset does not mediate the relationship between proactive personality and creativity.

For future directions, researchers could work on exploring other variables that may be conducive to the enhancement of creativity, such as feedback-seeking behavior and perseverance of effort. What’s more, regarding the age distribution of the participants, the present study only gives attention to undergraduate learners. Since the degree of golden mean thinking might vary from ages, we expect more studies to be done in the context of primary school, high schools and graduate educations. In the same light, it is also recommended that future researcher test the present research model in other Asian countries and even Western countries, which may help produce more culture-specific insights both for research purpose and practical purpose. Finally, as the present research have already adopted a quantitative method to investigate the intercorrelations among the mentioned variables, future research can choose qualitative ways such as using semi-structural interviews for more in-depth investigation into the proposed model.

## Implications

In terms of practical implications, these findings might be meaningful for educators working in the field of L3 pedagogy. First, given that proactive students often actively seek opportunities to help their learning and promote their ability to attain their goals (Li et al., 2010; Zhu et al., 2017), instructors should design some interesting programs to provide more opportunities for students to make FL presentations, such as role play in a real context and show photos or videos about relevant topics, to encourage less proactive L3 learners to participate in classroom activities (Wang, 2017; Zhu et al., 2017). Additionally, after class, teachers should design online discussion forums for students who are not brave enough in class to provide a platform for them to freely present their ideas or opinions.

Second, in relation to creativity, it is advisable to include creative teaching strategies into Chinese L3 instruction (Chen and Padilla, 2022). Students with higher willingness to exchange opinions and stronger perceptions of their own potential are more likely to be creative (Chen and Padilla, 2022). Hence, to induce their creative behaviors, teachers could encourage L3 learners to have dialog with partners or tell a story based on what they have learned and brainstormed in class, which motivates them to apply the learned information and strategies to actual use. Moreover, by comparing the similarities as well as the differences between L2 and L3 during their learning process, students may creatively develop a set of language-specific strategies for learning different languages, which can in the end do good to their creativity in the context of language learning (Chen and Padilla, 2022; Lei and Lei, 2022).

Third, a growth mindset motivates students to stand up against difficulties wholeheartedly with the assistance of their own efforts (Dweck, 1999), which can eventually be conducive to the cultivation of individuals’ creativity. Students who endorse a growth mindset perform better academically, especially when facing challenges (Haimovitz and Dweck, 2017). As such, the importance of promoting individuals’ growth mindset should not be neglected. Dweck (1999) mentioned that praising students for their efforts instead of intelligence significantly fosters growth mindsets in individuals. Therefore, it is suggested that teachers pay attention to students’ efforts in L3 learning and give timely compliments. This advice especially suits Asian Confucian cultures, in which effort is already highlighted in individuals’ learning process (Bai and Wang, 2020). Additionally, L3 teachers should foster students’ growth mindset by providing challenging learning opportunities such as reading circles, where each student has certain roles during reading or discussion and therefore involves actual usage of L3 instead of mechanical language practices (Begoray and Banister, 2008). Last, conducting process-oriented L3 teaching instead of focusing on students’ inborn abilities is more likely to enhance students’ malleable outlook on their intelligence (Haimovitz and Dweck, 2017), meaning that L3 teachers should also emphasize the process during which students acquire L3 skills and provide professional and specific guidance.

Fourth, teachers should introduce content involving Confucian philosophy and golden mean thinking in FL teaching to cultivate and strengthen students’ holistic thinking and big-picture perspective, thus guiding them to be able to integrate various factors while learning L3 (e.g., balance time in learning L1, L2, and L3 and combining cultures and languages). Moreover, given that golden mean thinking is also unconsciously influenced through individuals’ interactions with their teachers and peers (Liu, 2021), teachers should encourage students to evolve in cooperative learning to develop their proactive personality and creativity in a collaborative manner.

## Data availability statement

The raw data supporting the conclusions of this article will be made available by the authors, without undue reservation.

## Ethics statement

Ethical review and approval was not required for the study on human participants in accordance with the local legislation and institutional requirements. Written informed consent from the patients/participants or patients/participants legal guardian/next of kin was not required to participate in this study in accordance with the national legislation and the institutional requirements.

## Author contributions

FL: conceptualization, supervision, and revision. LL: review and revision. WD and YD: project organization. WD, YD, YG, and RL: data collection, writing-original draft, and editing. WD, YD, and YG: methodology and data analysis. All authors contributed to the article and agreed on the submitted version.
